# Thin film flow and heat transfer of Cu-nanofluids with slip and convective boundary condition over a stretching sheet

**DOI:** 10.1038/s41598-022-18049-3

**Published:** 2022-08-22

**Authors:** Azeem Shahzad, Fakhira Liaqat, Zaffer Ellahi, Muhammad Sohail, Muhammad Ayub, Mohamed R. Ali

**Affiliations:** 1 Basic Sciences Department, University of Engineering and Technology, Taxila, 47050 Pakistan; 2 Institute of Mathematics, Khwaja Fareed University of Engineering and Information Technology, Rahim Yar Khan, 64200 Pakistan; 3 Department of Mathematics, COMSATS University, Islamabad, Abbottabad Campus, Abbottabad, Pakistan; 4 Faculty of Engineering and Technology, Future University, Cario, Egypt; 5 Department of Mathematics, Benha Faculty of Engineering, Benha University, Benha, Egypt

**Keywords:** Mathematics and computing, Nanoscience and technology

## Abstract

The flow and heat transfer in thin film of Cu-nanofluid over a stretching sheet by considering different shape factors (platelets, blades, bricks, sphere and cylinder) along with slip and convective boundary conditions is investigated. The governing partial differential equations are converted to nonlinear ordinary differential equations by means of suitable similarity transformation and then solved by using BVP4C in MATLAB. The physical significance of various parameters on velocity and temperature profiles are investigated and provided in the form of table and also presented graphically. It is noted that the Platelet-shaped nanoparticles has the highest heat transfer rate as compare to other particle’s shapes.

## Introduction

Nanometer-sized metal particles have been manufactured using new technologies, leading in the development of a new class of fluid known as nanofluids. The mixing of extremely fine metallic particles (or smaller) in a saturated liquid, referred to as nanoparticles, is referred to as two-phase mixture. These fluids have a thermal conductivity that is significantly larger than that of the base fluid^1–3^. Nanofluids have been discovered to have improved thermal physical properties when compared to basic fluids. Nanofluids offer an alternative heat transfer medium, particularly at the micro and nanoscaled, where large heat fluxes are required. Despite their extraordinary potential and qualities, these exceedingly unique fluids are still in their infancy. Much of the early and scant experimental effort focused the determination of nanofluids' effective heat conductivity and dynamic viscosity^4,5^. Xuan and Roetzel^6^ studied the heat transport capacity of a nanofluid that changes due to the suspension of ultrafine particles, revealing a significant potential for improving heat transmission.

Copper nanoparticles made of metals have a wide range of applications in heat transfer, electronics, medicine, optics, and the production of antibacterial agents, nanofluids, and lubricants, among others. Due to the high reactivity of copper nanoparticles, oxidation occurs easily. Stabilization of pure copper metal nanoparticles is not achievable. Copper undergoes changes in its structural and thermal properties when it is oxidized to copper oxide. To avoid oxidation, a protective coating on the nanoparticles is utilized. The metallic particles are encapsulated using a variety of inorganic or organic chemicals^7,8^.

Heat transfer occurs naturally around us as a result of temperature differences, a process called natural convection. For example, sunlight heats water in rivers and on land. Additionally, fires, tectonic plates, and volcanic hot air are also instances of natural convective heat^9–11^. Sohail and Naz^12^ used Cattaneo-Christov theory to analyze the Sutterby nanofluid across a stretched cylinder. They discovered that increasing the magnetic parameter increases the temperature and concentration of the fluid, while having the opposite effect on velocity.

The phrase 'thin film' is frequently used to refer to flow that is defined by the fact that the flow domain in one dimension is significantly smaller than the flow domain in the other (one or two) dimensions. Flow difficulties in thin films are of critical practical importance. The notion of thin film is applied in a wide variety of industrial processes, including lubrication, surface coating, cooling of heat exchanger fins, and contact lens movement^13–15^. Khaled and Vafai^16^ examined the flow and temperature distribution within thin films by taking internal and exterior pressure pulsations into account. Bilal et al.^17^ used boundary layer theory to examine the heat transport and MHD Darcy-Forchheimer flow of a Sutterby fluid past a linearly stretched boundary. They noticed that when slip increases, magnetic parameters diminish the velocity profile, and a similar trend is evident for fluid temperature as thermal relaxation and slip parameters increase. Marzougui et al.^18^ investigated the formation of entropy in convective $Cu/{H}_{2}O$ nanofluid flow in a cavity containing Chamfers when a magnetic field was applied. They concluded that increasing the Hartman number decreases viscosity and thermal irreversibility.

Numerous researchers have discussed heat transfer analysis for thin film flow caused by stretching phenomena in non-Newtonian fluids, taking into account various effects such as magnetic field and porous medium, for both steady and unsteady flows; see, for example^19–32^, and the references therein. Tiwari and Das^32^ investigated the effect of relevant parameters on the heat transmission characteristics of nanofluids inside a heated square form cavity with a two-sided lid. They discovered that by incorporating nanoparticles into a base fluid, they were able to increase the base fluid's heat transfer capacity. Additionally, when solid volume fraction is used, the difference in the average Nusselt number is nonlinear. Naseem et al.^33^ investigated the three-dimensional flow properties and heat transfer of a $TiO_{2} {-}Cu/water$ nanofluid travelling across a bidirectional surface using the Tiwari and Das model and the nanofluid's thermophysical parameters. Additionally, higher Eckert and Prandtl values indicate a lower thermal profile. Shahrestani et al.^34^ examined laminar ${Al}_{2}{O}_{3}$/water nanofluid flow with a constant heat flux to the outer wall of an axisymmetric microchannel while maintaining adiabatic conditions at both ends. This was accomplished by taking only half of the axisymmetric microchannel into account and rotating the domain around its axis. They argued that raising the entrance velocity increases the rate of viscous dissipation and raises the temperature of the wall compared to the fluid's bulk temperature. Thus, the thermal behaviour of the fluid in microchannel is markedly different than in microchannel. Waini et al.^35^ examined the flow of a nanofluid toward a shrinking cylinder of ${Al}_{2}{O}_{3}$ nanoparticles. They determined that the first solution is stable. Afridi et al.^36^ investigated the flow and heat transfer properties of traditional and hybrid nanofluids. They investigated the development of entropy in conventional and hybrid nanofluid flows. Both nanofluids were considered to flow with heat dissipation along a narrow needle. They discovered that the hybrid nanofluid exhibited a more elevated temperature profile than the conventional nanofluid. Abbas et al.^37^ used a horizontal Riga plate to investigate the production of entropy in viscous nanofluids. Shankaralingappa et al.^38^ discussed the flow, heat and mass transfer by taking nonlinear fluid flow over stretching sheet by using Cattaneo–Christov heat flux model. The concentration profile drops as the thermophoretic and chemical reaction rate parameters increase in value. Many researcher work on stretching surfaces and analyze the different effects which can be seen in^38–45^.

To the authors' knowledge, no study has been conducted to investigate the effect of various shapes of Cu-nanofluid, such as sphere, cylinder, platelet, blade, and brick, on thin film flow and heat transfer over a stretching sheet with magnetic effect and convective boundary condition and partial slip, using water as the base fluid. The effect on temperature and velocity distributions of physical quantities, like, the volume fraction $\varphi$ of nanofluids the unsteadiness parameter $S$, the Prandtl number $\left( {Pr} \right)$, is carried out.

## Mathematical method and formulation

**Figure 1:**
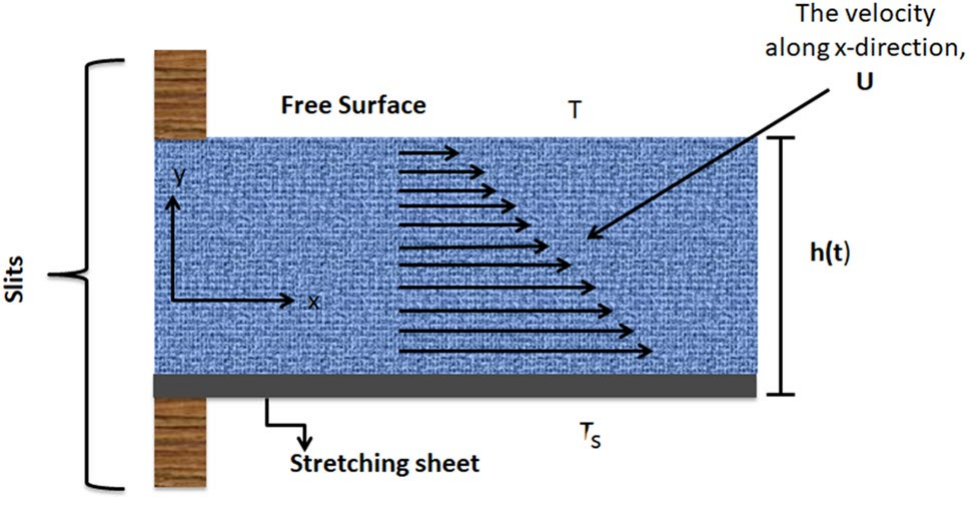
The flow of nanofluid composed by multi-nanomaterial.

In this investigation a 2-D unsteady, incompressible, a liquid thin film flow of $Cu/H_{2} O$ nanofluid past a stretchable sheet which is positioned along x-axis is considered as shown in Fig. 1. Thin film flow emerges because of the stretching of sheet. Thickness of the liquid film is $h\left( t \right)$, while the surface temperature and velocity of the stretching sheet, denoted by $U_{w}$ and $T_{s}$ respectively. The horizontal velocity is $U_{w} \left( {x,t} \right) = \frac{bx}{{1 - \alpha t}}$, ($a$ and $b$ are constants) and the wall temperature distribution is determined by:

$$ T_{s} = T_{o} - T_{r} \left( {\frac{{bx^{2} }}{{2\nu_{f} }}} \right)(1 - \alpha t)^{{ - \frac{3}{2}}} $$

The constant reference temperature and the temperature of the slit are respectively given as $T_{r}$ and $T_{0}$, whereas, $v_{f}^{*}$ is the kinematics viscosity of fluid. The perpendicular magnetic field (uniform) of strength $B\left( t \right) = \frac{{B_{0} }}{{\sqrt {1 - \alpha t} }}$ to the stretching layer is applied.

By using the Tiwari and Das model^26^ for nanofluid the governing continuity, momentum, and energy equations are given as

1 $$ \frac{{\partial u_{1}^{*} }}{\partial x} + \frac{{\partial u_{2}^{*} }}{\partial y} = 0, $$

2 $$ \frac{{\partial u_{1}^{*} }}{\partial t} + u_{1}^{*} \frac{{\partial u_{1}^{*} }}{\partial x} + u_{2}^{*} \frac{{\partial u_{1}^{*} }}{\partial y} = \frac{{\mu^{*}_{nf} }}{{\rho^{*}_{nf} }}\frac{{\partial^{2} u_{1}^{*} }}{{\partial^{2} y}} - \frac{{\sigma^{*}_{nf} }}{{\rho^{*}_{nf} }}B(t)^{2} u_{1}^{*} , $$

3 $$ \frac{{\partial T^{*} }}{\partial t} + u_{1}^{*} \frac{{\partial T^{*} }}{\partial x} + u_{2}^{*} \frac{{\partial T^{*} }}{\partial y} = \alpha^{*}_{nf} \frac{{\partial^{2} T^{*} }}{{\partial^{2} y}} + \frac{{\mu_{nf} }}{{\rho^{*}_{nf} C^{*}_{p} }}\left( {\frac{{\partial u_{1}^{*} }}{\partial y}} \right)^{2} , $$

where $u_{1}^{*} \left( {x,y,t} \right)$ and $u_{2}^{*} \left( {x,y,t} \right),$ are velocities and $T^{*} \left( {x,y,t} \right)$ represents the temperature. For present analysis, the boundaries conditions are

4 $$ u_{1}^{*} = U_{w} + A\nu^{*}_{f} \frac{{\partial u^{*} }}{\partial y},u_{2}^{*} = \frac{{dh^{*} }}{dt}, - k^{*}_{nf} \frac{{\partial T^{*} }}{\partial y} = h^{*}_{f} \left( {T_{ \circ } - T^{*} } \right),\,\,{\text{ at y}} = 0, $$

5 $$ \frac{{\partial u_{1}^{*} }}{\partial y} = \frac{{\partial T^{*} }}{\partial y} = 0, u_{2}^{*} = \frac{{dh^{*} }}{dt}, \,\,{\text{ at y}} = h^{*} \left( t \right), $$

where slip parameter and convective heat transfer coefficients of proportionality are $A$ and $h^{*}_{f}$ respectively. Thermo-physical properties such as $\mu^{*}_{nf}$, $\sigma^{*}_{nf}$, $\rho^{*}_{nf}$, $( {\rho^{*} C^{*}_{p} })_{nf}$, and $\alpha^{*}_{nf}$ are dynamic viscosity, electrical conductivity, density, and heat capacity, and diffusivity of the nanofluid respectively, mathematically given by^28,29^

6 $$ \begin{aligned} \alpha^{*}_{nf} & = \frac{{k^{*}_{nf} }}{{( {\rho^{*} C^{*}_{p} })_{nf} }}, \rho^{*}_{nf} = \left( {1 - \varphi } \right)\rho^{*}_{f} + \varphi \rho^{*}_{s} , \mu^{*}_{nf} \\ & = \mu^{*}_{f} \left( {1 + A^{*}_{1} \varphi + A^{*}_{2} \varphi^{2} } \right), \\ \sigma^{*}_{nf} & = \sigma^{*}_{f} \left( {1 - \varphi } \right)\sigma^{*}_{f} + \varphi \sigma^{*}_{s} , \left( {\rho^{*} C_{p}^{*} } \right)_{nf} \\ \, &= \left( {1 - \varphi } \right)( {\rho^{*} C^{*}_{p} })_{f} + \varphi ( {\rho^{*} C^{*}_{p} } )_{s} , \\ \end{aligned} $$

and

7 $$ \frac{{k^{*}_{nf} }}{{k^{*}_{f} }} = \left[ {\frac{{k^{*}_{s} + \left( {m - 1} \right)k^{*}_{f} + \left( {m - 1} \right)\left( {k^{*}_{s} - k^{*}_{f} } \right)\varphi }}{{k^{*}_{s} + \left( {m - 1} \right)k^{*}_{f} + \left( {k^{*}_{s} - k^{*}_{f} } \right)\varphi }}} \right], $$

where the volume fraction of the nanofluid is denoted by $\varphi$. The viscosity enhancement heat capacitance coefficients are $A^{*}_{1}$, $A^{*}_{2}$ and the heat power is expressed by $( {\rho^{*} C^{*}_{p} } )_{nf}$. Furthermore, $k^{*}_{s}$ and $m$, are thermal conductivity and size of the nanoparticle while thermophysical properties of base fluid, nanofluid, and nanoparticles are respectively displayed by subscripts $f, nf, {\text{and}}\, s$. In addition, the thermophysical properties of base fluid and nanoparticle are shown in Table 1 and properties regarding shape factors are mentioned in Table 2. Defining transformations of resemblance, as

8 $$  T_{s}  = T_{ \circ }  - T_{r} \left( {\frac{{bx^{{*2}} }}{{2\nu _{f} }}} \right)(1 - \alpha t)^{{ - \frac{3}{2}}} \theta \left( \eta  \right),~\eta  = \left( {\frac{b}{{\nu ^{*} _{f} \left( {1 - \alpha t} \right)}}} \right)^{{\frac{1}{2}}} ,\psi  = \left( {\frac{{b\nu ^{*} _{f} }}{{1 - \alpha t}}} \right)^{{\frac{1}{2}}} xf\left( \eta  \right),  $$

where stream function $\psi$ determine the pattern of flow and is defined as $u_{1}^{*} = \frac{\partial \psi }{{\partial y}}$ and $u_{2}^{*} = - \frac{\partial \psi }{{\partial x}}$, so that equation of continuity is satisfied identically. By substituting the above defined dimensionless variables Eq. (8) into Eqs. (2–3), the following nonlinear ODEs are obtained. Also by using (8) in (4, 5) the transformed boundary conditions becomes

9 $$ \varepsilon_{1} f^{\prime\prime\prime}\left( \eta \right) - \varepsilon_{3} Mf^{\prime}\left( \eta \right) + \left[ {f\left( \eta \right)f^{\prime\prime}\left( \eta \right) - f^{{^{{\prime}{2}} }} \left( \eta \right) - S\left( {f^{\prime}\left( \eta \right) + \frac{\eta }{2}f^{\prime\prime}\left( \eta \right)} \right)} \right] = 0, $$

10 $$ \frac{{\varepsilon_{2} }}{Pr}\theta^{\prime\prime}\left( \eta \right) + \varepsilon_{1} Ecf^{{\prime\prime}{2}} \left( \eta \right) + \left[ {f\left( \eta \right)\theta^{\prime}\left( \eta \right) - 2\theta \left( \eta \right)f^{\prime}\left( \eta \right) - \frac{S}{2}\left( {3\theta \left( \eta \right) + + \eta \theta^{\prime}\left( \eta \right)} \right)} \right] $$

and

11 $$\begin{gathered}   f\left( 0 \right) = 0,\;f^{\prime}\left( 0 \right) = 1 + Kf^{\prime\prime}\left( 0 \right),\;\theta ^{\prime}\left( 0 \right) =  - \frac{{k_{f} }}{{k_{{nf}} }}\gamma \left( {1 - \theta \left( 0 \right)} \right), \hfill \\   f\left( \beta  \right) = \frac{{S\beta }}{2},\;f^{\prime\prime}\left( \beta  \right) = 0,\;\theta ^{\prime}\left( \beta  \right) = 0. \hfill \\  \end{gathered}  $$

**Table 1:** Thermophysical characteristics of $H_{2} O/Cu$^13^.

Nanoparticle and Base fluid	Thermal Conductivity $\left( {\text{W/mK}} \right)$	Density $\left( {{\rm Kg/m}^{3}} \right)$	Electrical Conductivity $\left( {\text{S/m}} \right)$	Specific heat $\left( {\text{J/KgK}} \right)$
$Cu$	401	8933	59.6	385
$H_{2} O$	0.613	97.19	5.50	4179

**Table 2:** Heat capacitance coefficients, size, and nanoparticle’s shapes^14^.

Nanoparticle’s shape	$A1$	$A2$	$m$
Platelet	$37.1$	$612.6$	$5.72$
Cylinder	$13.5$	$904.4$	$4.82$
Blade	$14.6$	$123.3$	$8.26$
Brick	$1.9$	$471.4$	$3.72$
Sphere	$2.5$	$6.5$	$3.0$

The dimensionless constants $K = A\sqrt {\frac{{\nu^{*}_{f} U_{w} }}{x}}$, $E_{c} = \frac{{U_{w}^{2} }}{{C^{*}_{p} \left( {T_{s} - T^{*} } \right)}}$, $M = \frac{{\beta_{ \circ }^{2} \sigma^{*}_{f} }}{{b\rho^{*}_{nf} }}$, $P_{r} = \frac{{\rho^{*} (C^{*}_{p} )_{f} \nu^{*}_{f} }}{{k^{*}_{f} }}$ and $S = \frac{a}{b}$ are slip parameter, Eckert number, magnetic parameter, Prandtl number and unsteadiness parameter respectively. Additionally,$\varepsilon_{i}$, $i = 1,...,3$ are constants and described as

12 $$ \varepsilon_{1} = \frac{{1 + A^{*}_{1} \varphi + A^{*}_{2} \varphi^{2} }}{{1 - \varphi + \varphi \left( {\frac{{\rho^{*}_{s} }}{{\rho^{*}_{f} }}} \right)}},\varepsilon_{2} = \frac{{\frac{{k^{*}_{nf} }}{{k^{*}_{f} }}}}{{1 - \varphi + \varphi \frac{{(\rho^{*} C^{*}_{p} )_{s} }}{{(\rho^{*} C^{*}_{p} )_{f} }}}},\varepsilon_{3} = \frac{{1 - \varphi + \varphi \left( {\frac{{\sigma^{*}_{s} }}{{\sigma^{*}_{f} }}} \right)}}{{1 - \varphi + \varphi \left( {\frac{{\rho^{*}_{s} }}{{\rho^{*}_{f} }}} \right)}}, $$

where $\varphi^{*}$ is the solid volume-fraction. Skin shear stress and heat transfer coefficient are described as

13 $$ \begin{gathered} C_{f} = \frac{{\tau_{w} }}{{\rho_{f}^{*} U_{w}^{2} }}\,\, {\text{and}} \,\,Nu = \frac{{x^{*} q_{w} }}{{k_{f}^{*} \left( {T_{s} - T_{0} } \right)}}, \hfill \\ {\text{where}} \hfill \\ \tau_{w} = \mu_{nf}^{*} \left[ {\frac{{\partial u_{1}^{*} }}{{\partial y^{*} }}} \right]_{{y^{*} = 0}} , q_{w} = - k_{nf}^{*} \left[ {\frac{{\partial T^{*} }}{{\partial y^{*} }}} \right]_{{y^{*} = 0}} . \hfill \\ \end{gathered} $$

The non-dimensional form of Eq. (13) with respect to transformed variables is

14 $$ C_{f} = \frac{{\left( {1 + A^{*}_{1} \varphi + A^{*}_{2} \varphi^{2} } \right)}}{{Re^{\frac{1}{2}} }}f^{\prime\prime}\left( 0 \right),{ }{\text{Re}}^{{\frac{ - 1}{2}}} Nu = - \frac{{k^{*}_{nf} }}{{k^{*}_{f} }}\theta^{\prime}\left( 0 \right). $$

## Solutions of the problem

By using the similarity transformation, non-linear PDEs with boundary conditions are transformed into nonlinear ODEs. Here, these non-linear ODEs have been reduced to first order ordinary differential equations as

15 $$ y_{1} = f,\;y_{1}^{\prime } = y_{2} ,\,\;y_{2}^{^{\prime}} = y_{3} , $$

16 $$ y_{3}^{^{\prime}} = \frac{{\varepsilon_{3} }}{{\varepsilon_{1} }} + My_{2} - \frac{1}{{\varepsilon_{1} }}\left[ {y_{1} y_{3} - y_{2}^{2} - S\left( {y_{2} + \frac{\eta }{2}y_{3} } \right)} \right], $$

17 $$ \begin{gathered} \theta = y_{4} ,y_{4}^{^{\prime}} = y_{5} \hfill \\ y_{5}^{^{\prime}} = - \frac{{\varepsilon_{1} }}{{\varepsilon_{2} }}PrEcy_{3}^{2} - \frac{1}{{\varepsilon_{2} }}\left[ {y_{1} y_{5} - 2y_{4} y_{2} - \frac{S}{2}\left( {3y_{4} + \eta y_{4} } \right)} \right], \hfill \\ \end{gathered} $$

18 $$ y_{1} \left( 0 \right) = 0,y_{2} \left( 0 \right) = 1 + Ky_{3} \left( 0 \right),y_{5} \left( 0 \right) = - \gamma \left( {1 - y_{4} \left( 0 \right)} \right), $$

19 $$ y_{2} \left( \beta \right) = \frac{S\beta }{2},y_{3} \left( \beta \right) = 0,y_{5} \left( \beta \right) = 0. $$

**Figure 2:**
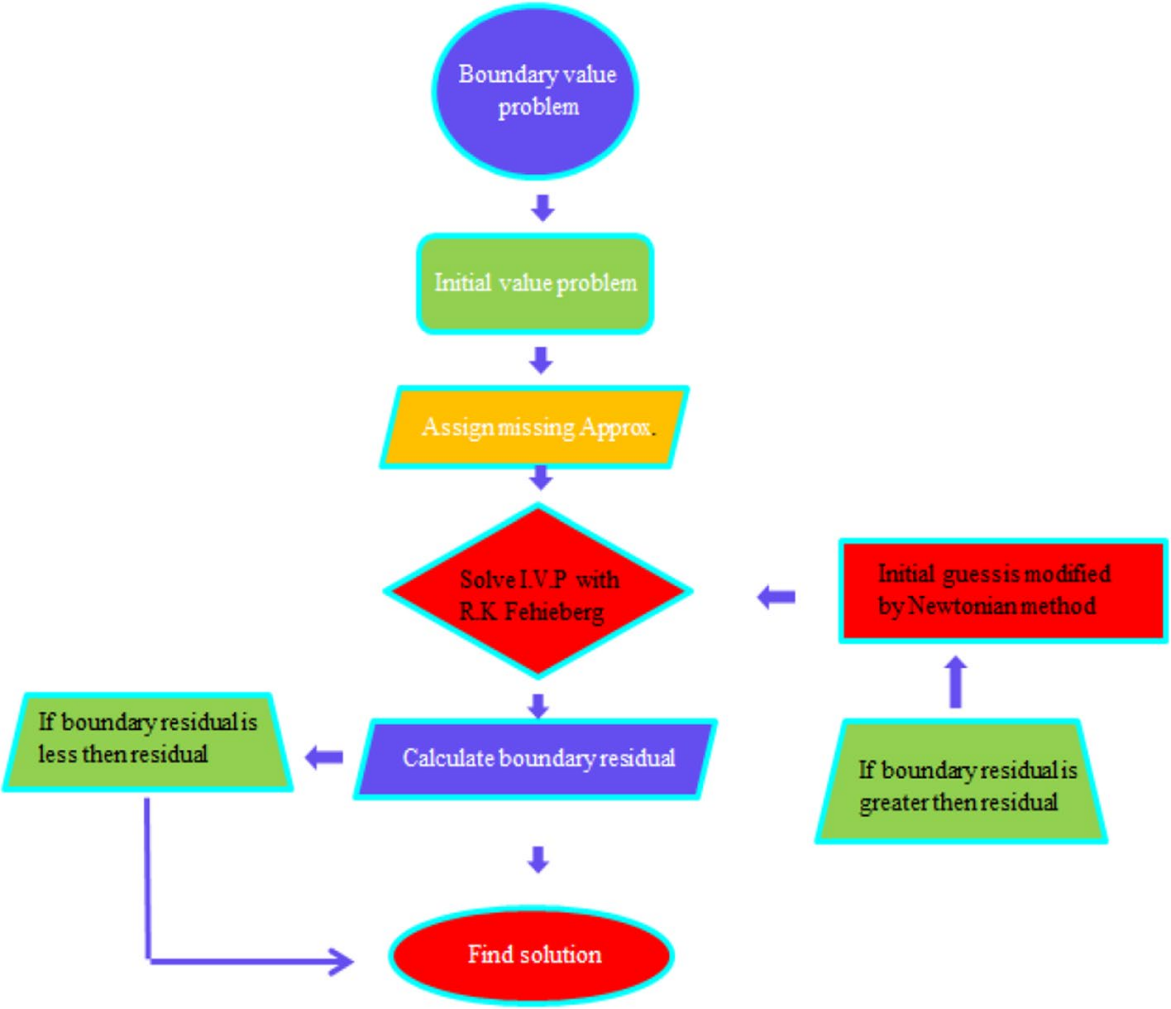
Flow chart related to numerical scheme.

The extraneous condition $y_{2} \left( \beta \right) = \frac{S\beta }{2}$ is utilized to evaluate, which is achieved using a hit-and-trial approach. Using BVP4C in MATLAB, the coupled ODE system is solved for the known values of $S$ and $\beta .$ The flow chart is shown in Fig. 2.

## Numerical results and discussion

The physical flow quantities, Eckert number $\left( {Ec} \right)$, Prandtl number $(\Pr )$, Biot number $\left( \gamma \right)$, unsteadiness parameter $S$, and magnetic field strength $\left( M \right)$ are crucial in the temperature, velocity, and local heat transfer rate for the physical problem under consideration. In this section numerical results derived for solving Eqs. (9–11) with BVP4C approach. The change in different shapes of nanoparticles in nanofluid thin film over stretched layer influences both temperature and velocity profiles. The enhanced thermal conductivity of the base fluid due to increase in volume fraction of base fluid, nanoparticles boost the heat of the base fluid. The high concentration of nanoparticles in the thermal boundary layer on the wall side, which can be explained by nanoparticle migration, is one of the reasons for improved nanofluid heat transfer. It should also be observed that as the volume fraction grows, so does the thickness of the thermal boundary layer. The increase in shear stress and skin friction leads the nanofluids speed to drop towards the end.

**Figure 3:**
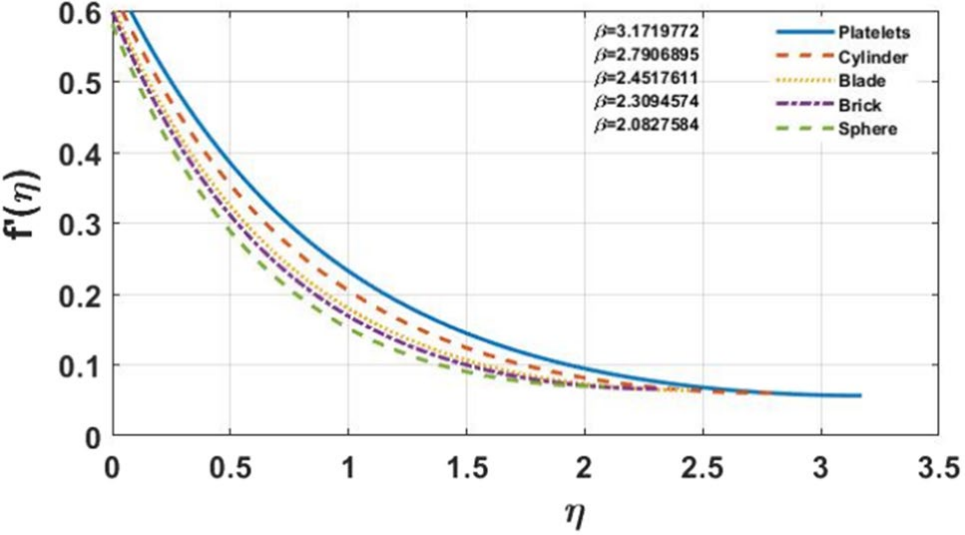
The impact of shape of nanoparticle on $f^{\prime}\left( \eta \right)$, for $\varphi = 0.02,S = 0.4,K = 0.5,M = 1.0.$

**Figure 4:**
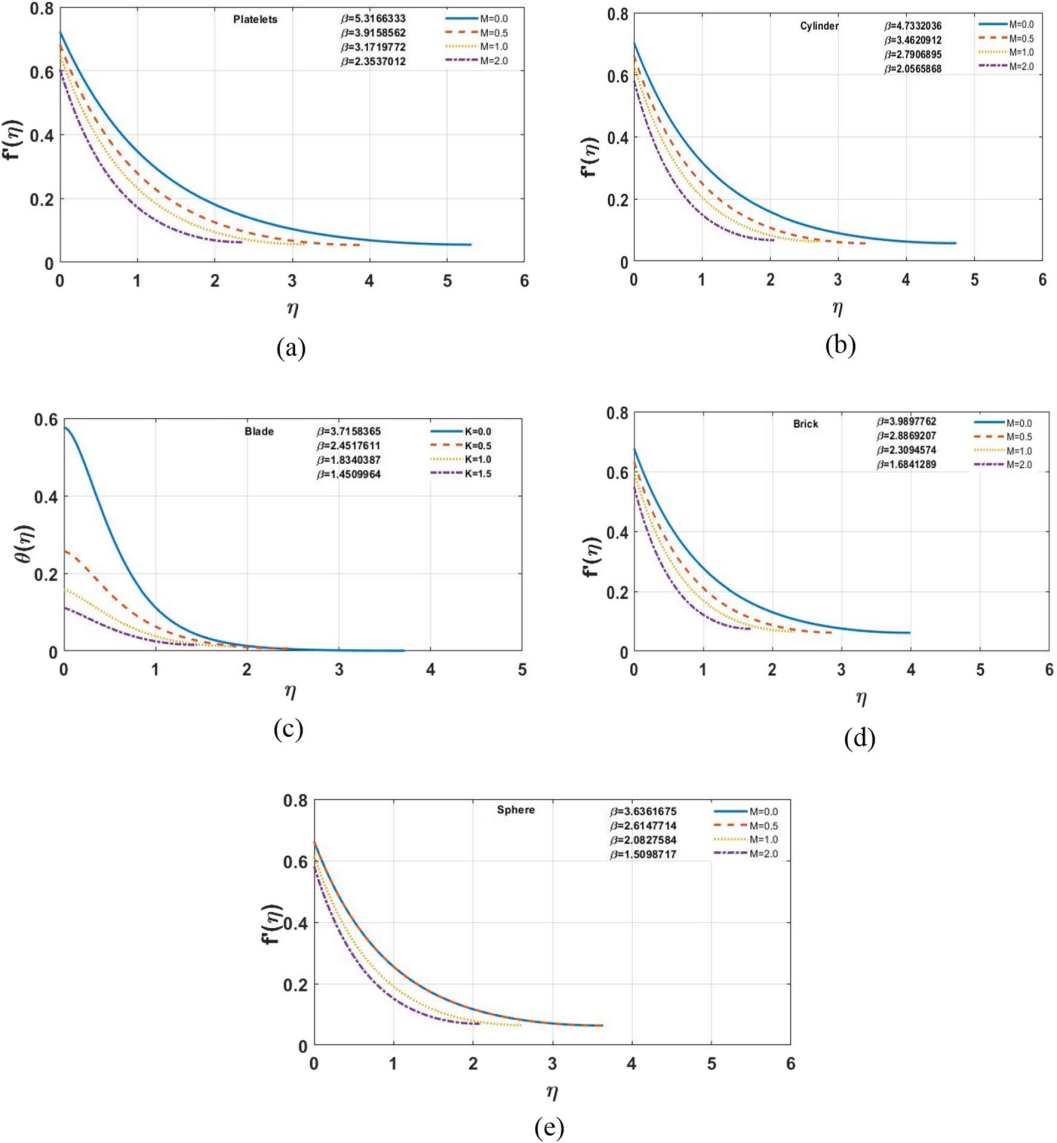
**(a–e)** Effects of Slip parameter M on the velocity profile for S = 0.4, K = 0.5 and φ = 0.02.

**Figure 5:**
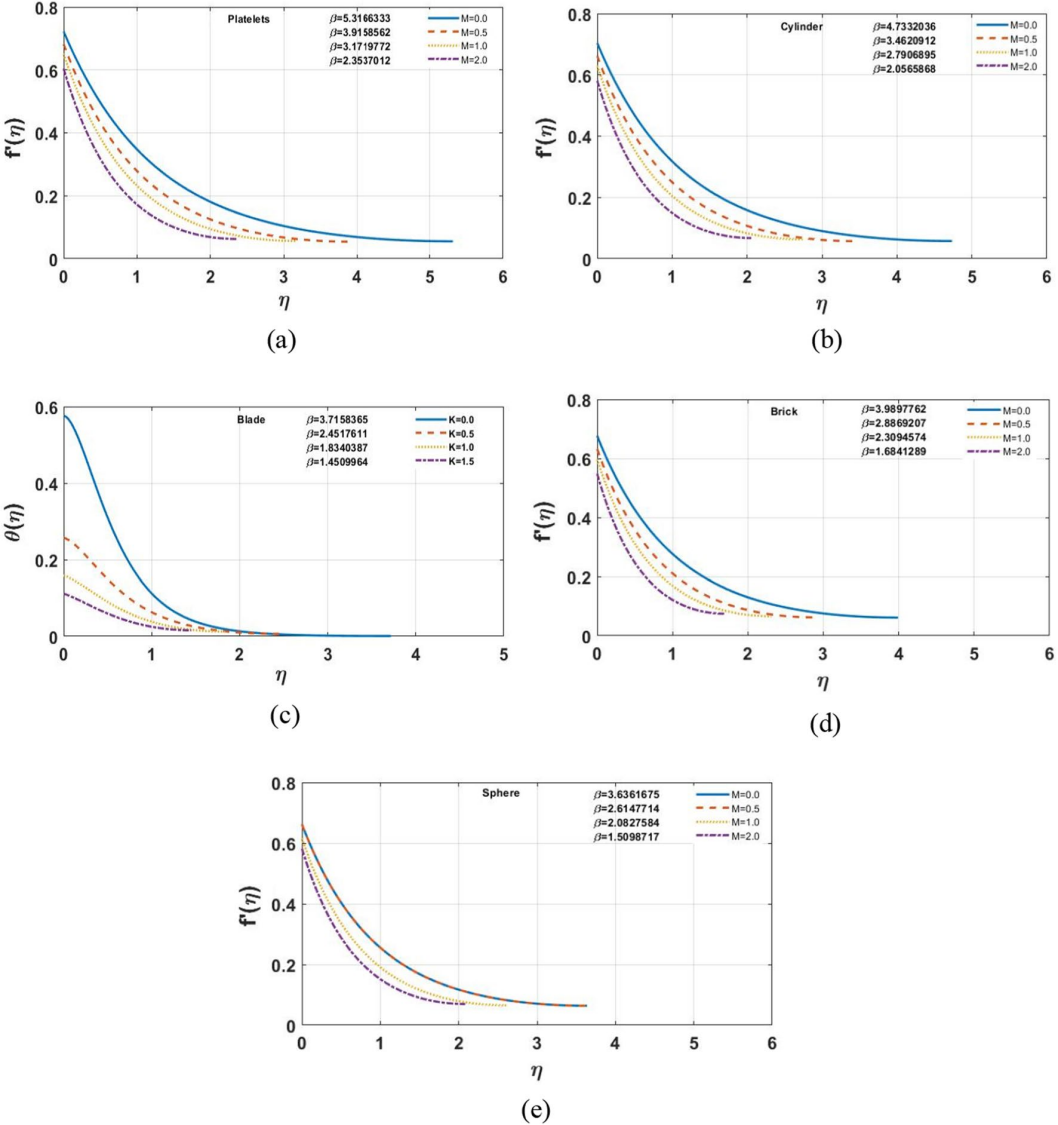
**(a–e)** The impact of $S$ on $\theta \left( \eta \right)$, for $S = 0.4,\,\,M = 1.0,\,\,\varphi = 0.02,\,\,\gamma = 0.1,\,\,Ec = 1.0,\,\,\Pr = 6.0.$

**Figure 6:**
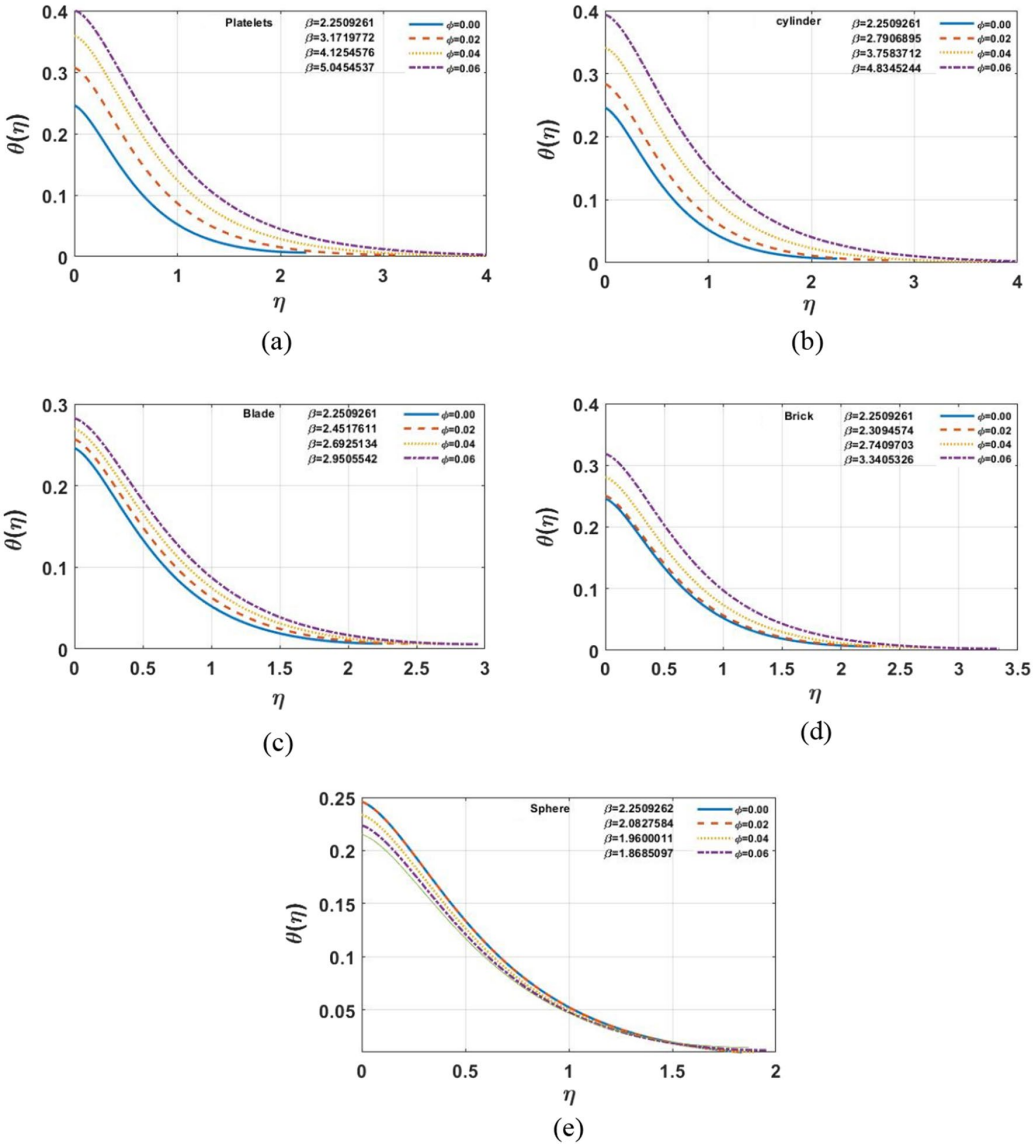
**(a–e)** The impact of $\varphi$ on $\theta \left( \eta \right)$, for $\Pr = 6.0,\,\,M = 1.0,\,\,S = 0.4,\,\,K = 0.5,\,\,Ec = 1.0,\,\,\gamma = 0.1$.

The impact of different shapes of nanoparticles on the film thickness $$\beta$$ of $$Cu$$ -nanofluids is shown in Fig. 3, while other physical parameters are held constant. The thickness of the film was substantially changed by varying the shapes of the nanoparticles. Although the fact that the film thickness value increases for platelet nano-sized particles and lowers for other forms of nanoparticles such as blade, cylinder, brick, and sphere comparatively. The impact of the magnetic parameter $$M$$ on different shapes of nanoparticles displayed in the Fig. 4a–e. It is observed that as the value of $$M$$ increases, the velocity profile for each multi-shape nanoparticle decreases. The impact of the slip parameter $$k$$ on different shapes of nanoparticles displayed in the Fig. 5a–e. It is observed that as the value of $$K $$ drops, the temperature profile for each multi-shape nanoparticle diminishes. With higher values of parameter $$K$$, the thickness of the film $$\beta$$ also decreases. Figure 6a–e shows that better results are obtained in the variation of $$\varphi ,$$ with a little change in temperature profile for spherical particles. Furthermore, as the thickness of the film $$\beta$$ grows, so does the volume-fraction $$\varphi$$ parameter.

**Figure 7:**
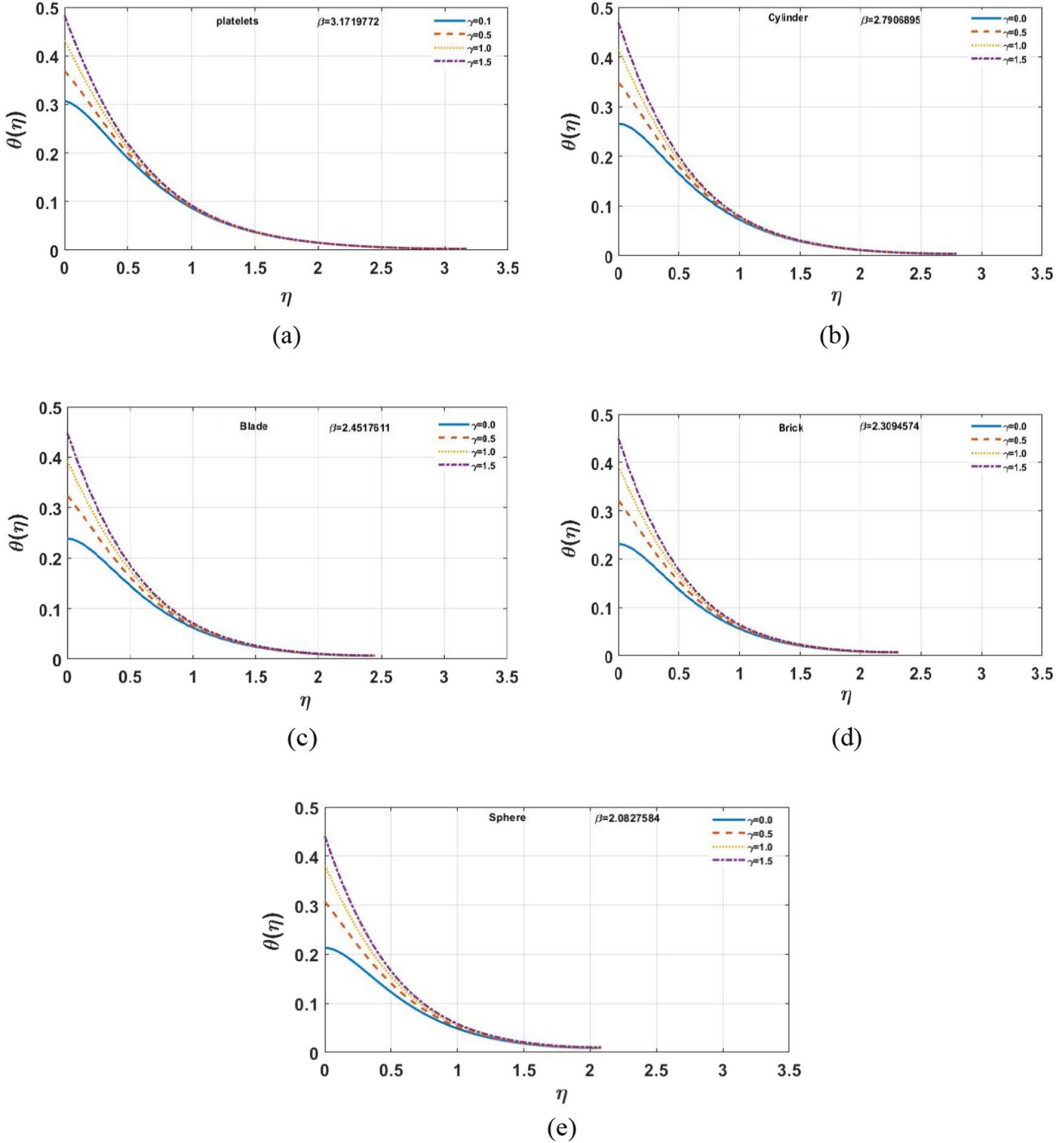
**(a–e)** The impact of $\gamma$ on $\theta \left( \eta \right)$ for $\varphi = 0.02$, $K = 0.5$, $\Pr = 6.0$, $Ec = 1.0$, $S = 0.4$, $M = 1.0$.

**Figure 8:**
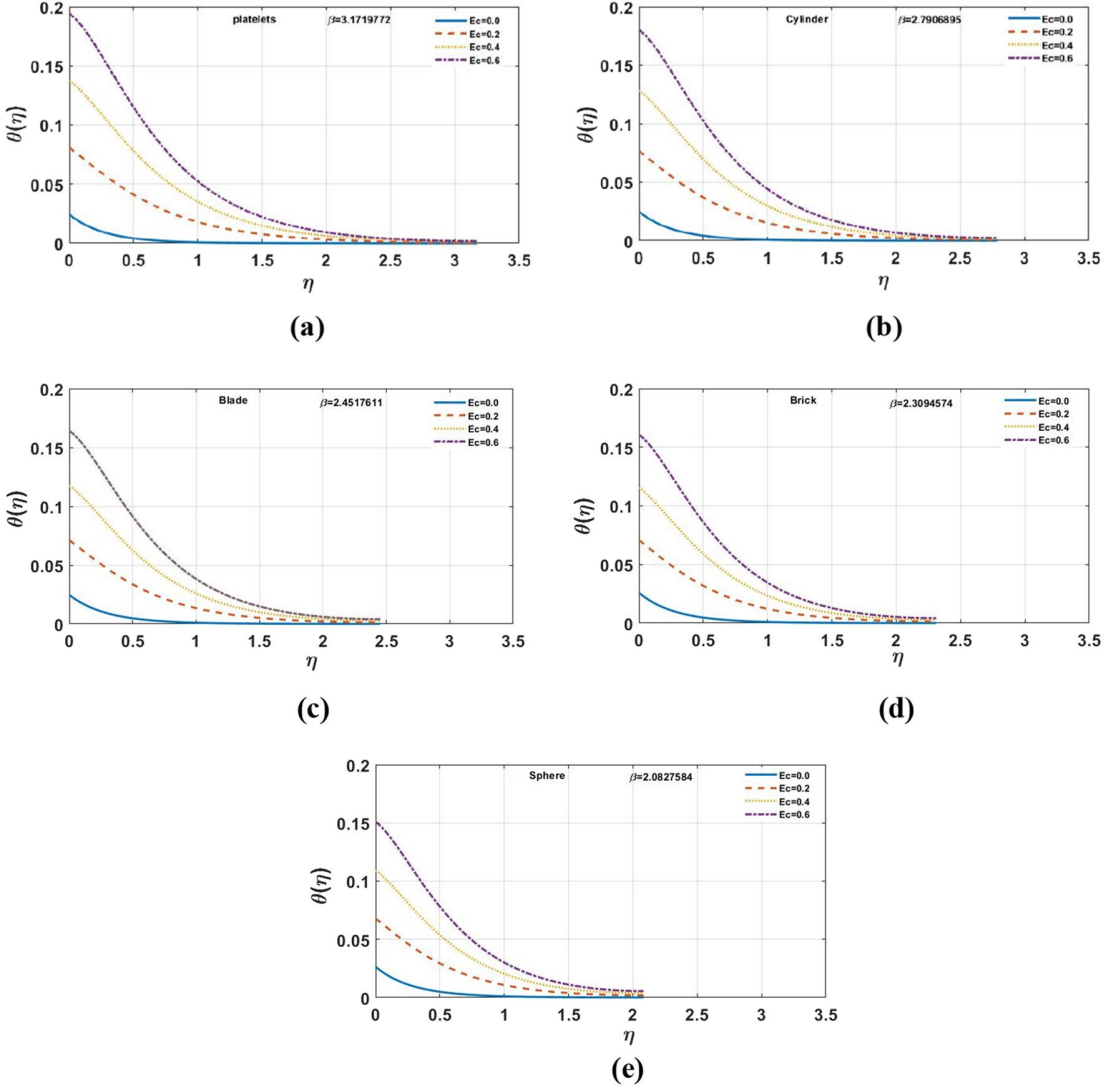
**(a–e)** The impact of $Ec$ on $\theta \left( \eta \right)$ for $S = 0.4$, $M = 1.0$, $K = 0.5$, $\varphi = 0.02$, $\gamma = 0.1$, $\Pr = 6.0.$

**Figure 9:**
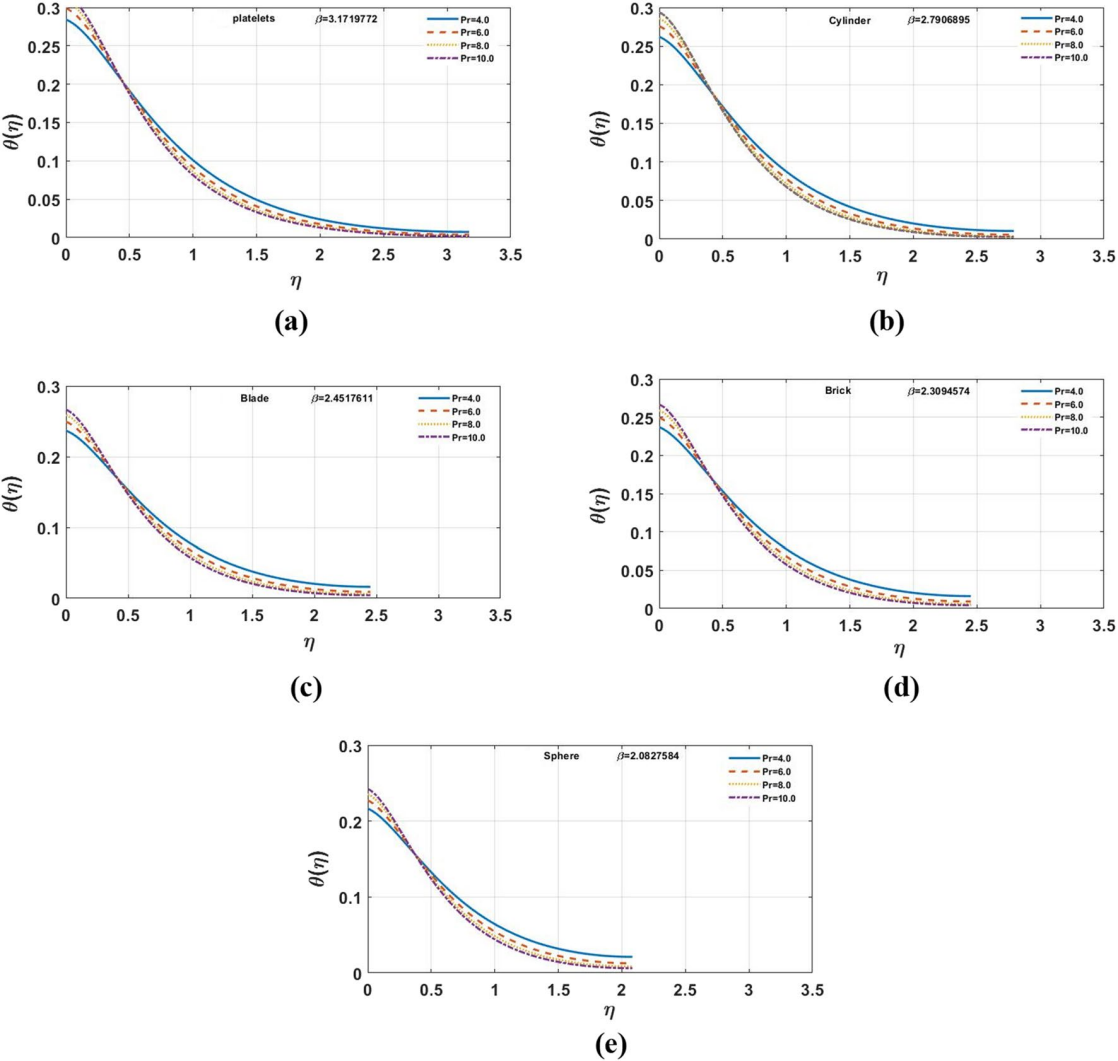
**(a–e)** The impact of $Pr$ on $\theta \left( \eta \right),$ for $M = 1.0$, $\varphi = 0.02,\gamma = 0.1$, $K = 0.5$, $S = 0.4$, $Ec = 1.0$.

**Figure 10:**
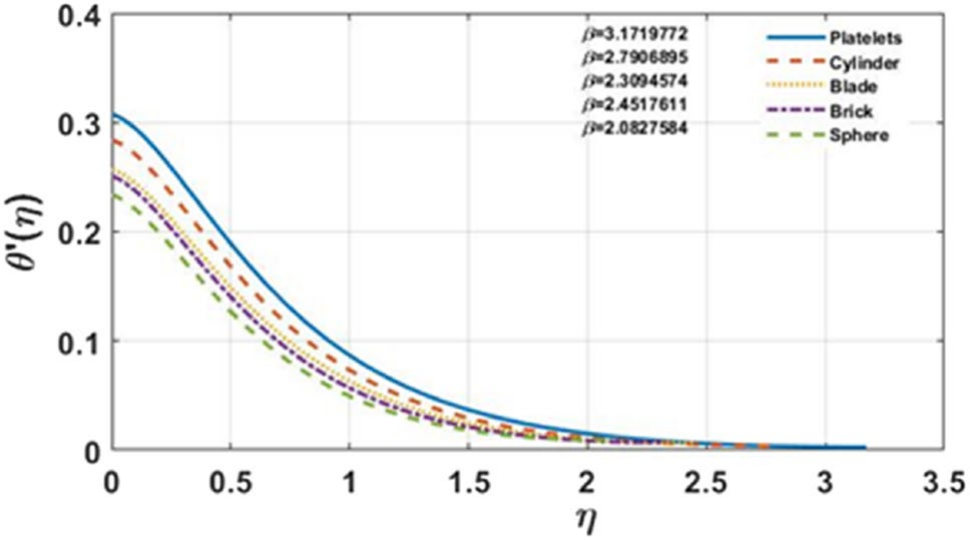
The influence of shape factor on temperature profile, for fixed values of $\varphi = 0.02,M = 1.0,\gamma = 0.1,K = 0.5,S = 0.4,\Pr = 6.0,Ec = 1.0$.

Figure 7a–e indicates influence of $\gamma$ on the temperature distribution. For the growing values of $\gamma$ steadily increase in results can be seen for multi-shape nano-size particles. Moreover, for increasing values of $\gamma$ temperature also increases. The Biot-number is the ratio of heat convection at the surface to conduction within the surface. As the thermal gradient is introduced to a surface, the temperature inside the surface changes dramatically, while and the surface heats/cools with time. The temperature profile is clearly affected when the value of $Ec$ grows, however, for brick and platelet shaped nanoparticles the temperature distribution is less effected as shown in Fig. 8a–e From physically point of view, Eckert number is based on viscous dissipation whereas concept of viscous dissipation is raised using term work done of particles in view of heat transfer phenomenon. So, an increment in Eckert number results an increment in viscous dissipation. It means that thermal layers are increased when Eckert number is increased. Furthermore, the velocity profile and film thickness increase for platelets and decreases for other nano sized particles. The increase in Prandtl number $Pr$ decreases the temperature profile shown in Fig. 9a–e. For the larger Prandtl number, the fluids retain weaker thermal diffusivity and conversely. Ratio among thermal and momentum layers makes a Prandtl number and measurement of thermal as well as momentum layers is analyzed using numerical values of Prandtl number. Thickness of thermal layers can be easily controlled by Prandtl number. The reduction in temperature profile produces due to the change in thermal diffusivity. In contrast to platelet shape nanoparticles, change is found for brick shape nanoparticles. In addition, for platelet shape nanoparticles the velocity distribution and the film thickness expedite while reverse behavior is observed for other nanoparticles. The temperature profile for different shapes of nanoparticles is shown in the Fig. 10. It is found that the platelet shape of nanoparticles is highly influential as compare to others.

The numerical results of dimensionless skin-friction coefficient for different shape of nanoparticles are given in Table 3. Decreasing values of skin-friction coefficient are found for increasing values of slip parameter $K$ and unsteadiness parameter $S$, whereas the reverse behaviour is noticed in case of magnetic parameter $M$ and volume-fraction parameter $\varphi .$

**Table 3:** The skin-friction coefficient's numerical values for multi-shaped nanoparticles.

Physical parameter				Platelets	Cylinder	Blade	Brick	Sphere
$S$	$M$	$K$	$\varphi$	$f{^{\prime}}{^{\prime}}(0)$				
0.4	–	–	–	1.386092	1.207706	1.050703	0.985285	0.881762
0.6	–	–	–	1.371608	1.214893	1.054369	0.987525	0.881803
0.8	–	–	–	1.397411	1.187585	1.026065	0.958923	0.852904
–	0.0	–	–	1.101854	0.964370	0.844057	0.792183	0.711687
–	0.5	–	–	1.262221	1.102173	0.961058	0.902181	0.808897
–	1.0	–	–	1.386092	1.207380	1.050703	0.985285	0.881762
–	2.0	–	–	1.572132	1.364654	1.182631	1.106975	0.987503
–	–	0.0	–	2.314394	2.097308	1.901517	1.814163	1.684479
–	–	0.5	–	1.386092	1.207706	1.050703	0.985285	0.881761
–	–	1.0	–	1.002118	0.859146	0.735391	0.684465	0.604718
–	–	1.5	–	0.785448	0.666745	0.565083	0.523579	0.459017
–	–	–	0.00	0.812143	0.812143	0.812143	0.812143	0.812143
–	–	–	0.02	1.386092	1.207706	1.050703	0.985285	0.881762
–	–	–	0.04	2.118136	1.091649	1.339250	1.365171	0.952327
–	–	–	0.06	2.974656	2.841773	1.673068	1.911689	1.024716

The thermal transfer rate is also measured and given in Table 4. The increase in Prandtl and Eckert numbers causes decay in the Nusselt number. For increasing value of Biot-number and unsteadiness parameter, increase in Nusselt number is also observed.

**Table 4:** The numerical results Nusselt number $-\theta {^{\prime}}(0)$ for multi-shape nanoparticles.

Physical parameter						Platelets	Cylinder	Blade	Brick	Sphere
$S$	$K$	$\Phi$	$Ec$	$Pr$	$\gamma$	$-\theta {^{\prime}}(0)$				
0.4	0.4	0.02	1.0	7.56	0.1	0.0692554	0.0716217	0.0743009	0.0749311	0.0766112
0.6	−	−	−	−	−	0.0731650	0.0755059	0.0781246	0.0787672	0.0804123
0.8	−	−	−	−	−	0.0780458	0.0803049	0.0827557	0.0833739	0.0848734
−	0.0	−	−	−	−	0.0382963	0.0399336	0.0423572	0.0428332	0.0437179
−	0.5	−	−	−	−	0.0692554	0.0716217	0.0743009	0.0749311	0.0766112
−	1.0	−	−	−	−	0.0801710	0.0820764	0.0841391	0.0846297	0.0858719
−	1.5	−	−	−	−	0.0857099	0.0872269	0.0888311	0.0891859	0.0900966
−	−	0.02	−	−	−	0.0692554	0.0716217	0.0743009	0.0749311	0.0766112
−	−	0.04	−	−	−	0.0640684	0.0658535	0.0730262	0.0718965	0.0776249
−	−	0.06	−	−	−	0.0599186	0.0606182	0.0717395	0.0681422	0.0784721
−	−	−	0.0	−	−	0.0975700	0.0975078	0.0975301	0.0974143	0.0973575
−	−	−	0.2	−	−	0.0919071	0.0923306	0.0928843	0.0929177	0.0932082
−	−	−	0.4	−	−	0.0862442	0.0871534	0.0882384	0.0884210	0.0890589
−	−	−	0.6	−	−	0.0805813	0.0819761	0.0835926	0.0839244	0.0849097
−	−	−	−	4.0	−	0.0715811	0.0737887	0.0763178	0.0768301	0.0783452
−	−	−	−	6.0	−	0.0701116	0.0724280	0.0750631	0.0756529	0.0772808
−	−	−	−	8.0	−	0.0690458	0.0714228	0.0741113	0.0747509	0.0764426
−	−	−	−	10.0	−	0.0682222	0.0706374	0.0733566	0.0740319	0.0757657
−	−	−	−	−	0.5	0.3156011	0.0325645	0.0338102	0.3395379	0.0346438
−	−	−	−	−	1.0	0.5682739	0.5850011	0.6078829	0.6078562	0.6189190
−	−	−	−	−	1.5	0.7751334	0.7964388	0.8281516	0.8252357	0.8388418

The comparison of obtained results with the available literature and given in Table 5 which shows that proposed scheme is accurate and convergent.

**Table 5:** The numerical results with the published work for $M,\,\,K, \mathrm{and\,\, \phi }=0$.

	Wang^47^		Abel et al.^48^		Li et al.^46^		Present	
*S*	$\beta$	$f^{\prime\prime}\left( 0 \right)$	$\beta$	$f^{\prime\prime}\left( 0 \right)$	$\beta$	$f^{\prime\prime}\left( 0 \right)$	$\beta$	$f^{\prime\prime}\left( 0 \right)$
0.4	5.12249	− 1.30778	4.98146	− 1.13409	4.98146	− 1.134	4.98147	− 1.13409
0.6	3.13125	− 1.19516	3.13171	− 1.19513	3.13192	− 1.195	3.13171	− 1.19512
0.8	2.15199	− 1.24579	2.15199	− 1.24580	2.15237	− 1.246	2.15202	− 1.24581
1.0	1.54362	− 1.27776	1.54362	− 1.27777	1.54359	− 1.278	1.54362	− 1.27777
1.2	1.12778	− 1.27918	1.12278	− 1.27917	1.12778	− 1.279	1.12778	− 1.27917
1.4	0.82103	− 1.23355	0.82210	− 1.23354	0.82103	− 1.234	0.82103	− 1.23354
1.6	0.57617	− 1.11494	0.57617	− 1.11494	0.57617	− 1.115	0.57617	1.114937
1.8	0.35639	− 0.86741	0.35639	− 0.86742	0.35639	− 0.8674	0.35639	0.867410

## Conclusion and key findings

A thermal transport analysis is recorded in nanofluids in the presence of convective boundary conditions and heat generation/absorption for multiple shapes of tiny particles. Over an unsteady thin film, the flow is carried out. The effects on the velocity and temperature behaviour of the main flow parameters are demonstrated.

- Prandtl and Eckert number decreases for slip parameter, but on the other hand unsteadiness parameter, Biot-number, and Nusselt number increases for slip parameter;
- Among the studied particle forms, the velocity of $Cu$ nanofluids consisting of platelet-shaped tiny particles is largest. The $Cu$ nano fluid's velocity profile approaches its minimum for nanoparticles of sphere-shape, while thermal conductivity shows a similar pattern;
- Additionally, as the Biot number $\gamma$ grows, the temperature $\theta (\eta )$ increases, whereas the value of film thickness $\beta$ increases for platelet-shaped nanoparticles and drops for cylinder, blade, brick, and sphere-shaped nanoparticles;
- For slip and unsteadiness parameters, the skin friction coefficient is reduced while the volume-fraction parameter is increased;
- When the slip parameter $K$ is increased the film thickness $\beta$ is getting reduced;
- Furthermore, for the increase in volume-fraction $\varphi$ the film thickness $\beta$ also increases.

## Data Availability

The datasets generated/produced during and/or analyzed during the current study/research are available from the corresponding author on reasonable request.

## List of symbols

$$u_{1}^{*} \left( {x,y} \right), u_{2}^{*} \left( {x,y} \right)$$: Velocities components(m/s)
$\beta$: Thickness of film (–)
$$b$$: Constants (S^−1^)
$u_{w}$: Velocity of surface (m/s)
$T_{s}$: Temperature of surface (K)
$$T_{r}$$: Reference temperature (K)
$$T_{o}$$: Slit's temperature (K)
$k^{*}_{f}$: Base fluid's thermal conductivity (W/K/m)
$k^{*}_{nf}$: Nanofluid's thermal conductivity (W/K/m)
$c^{*}_{p}$: Fluid's specific heat (J/kg/K)
$$Nu$$: Nusselt number (–)
$${\text{Re}}$$: Reynold number (–)
$$\Pr$$: Prandtl number (–)
$$B_{o}$$: Magnetic field
$$\eta$$: Similarity variable
$\varphi$: Nanoparticle's Volume fraction
$\alpha^{*}_{f}$: Thermal diffusivity of water
$\alpha^{*}_{nf}$: Thermal diffusivity of nanofluid
$\rho^{*}_{f}$: Density of base fluid (water)
$\rho^{*}_{nf}$: Density of nanofluid
$\mu^{*}_{f}$: Dynamic viscosity of water
$\mu^{*}_{nf}$: Dynamic viscosity of nanofluid
$\nu_{f}$: Kinematic viscosity of water
$\nu_{nf}$: Kinematic viscosity of nanofluid
$$\sigma^{*}_{f}$$: Electrical conductivity
$( {\rho^{*} C^{*}_{p} })_{nf}$: Heat capacity of nanofluid
