# Author Correction: Thin film flow and heat transfer of Cu-nanofluids with slip and convective boundary condition over a stretching sheet

**DOI:** 10.1038/s41598-022-20620-x

**Published:** 2022-09-20

**Authors:** Azeem Shahzad, Fakhira Liaqat, Zaffer Ellahi, Muhammad Sohail, Muhammad Ayub, Mohamed R. Ali

**Affiliations:** 1 Basic Sciences Department, University of Engineering and Technology, Taxila, 47050 Pakistan; 2 Institute of Mathematics, Khwaja Fareed University of Engineering and Information Technology, Rahim Yar Khan, 64200 Pakistan; 3 Department of Mathematics, COMSATS University, Islamabad, Abbottabad Campus, Abbottabad, Pakistan; 4 Faculty of Engineering and Technology, Future University, Cairo, Egypt; 5 Department of Mathematics, Benha Faculty of Engineering, Benha University, Benha, Egypt

Correction to: *Scientific Reports* 10.1038/s41598-022-18049-3, published online 22 August 2022

The original version of this Article omitted an affiliation for Mohamed R. Ali. The correct affiliations are listed below.

Faculty of Engineering and Technology, Future University, Cairo, Egypt.

Department of Mathematics, Benha Faculty of Engineering, Benha University, Benha, Egypt.

The original Article has been corrected.

